# Peer support for frequent users of inpatient mental health care in Uganda: protocol of a quasi-experimental study

**DOI:** 10.1186/s12888-019-2360-8

**Published:** 2019-11-29

**Authors:** Grace K. Ryan, Mauricia Kamuhiirwa, James Mugisha, Dave Baillie, Cerdic Hall, Carter Newman, Eddie Nkurunungi, Sujit D. Rathod, Karen M. Devries, Mary J. De Silva, Richard Mpango

**Affiliations:** 1 Department of Population Health, London School of Hygiene and Tropical Medicine, Keppel Street, WC1E 7HT, London, UK; 2 Butabika National Referral Hospital, Kampala, Uganda; 3 East London National Health Service Foundation Trust, London, UK; 4 Camden and Islington National Health Service Foundation Trust, London, UK; 5 Harvard T.H. Chan School of Public Health, Boston, MA USA; 6 Butabika Recovery College, Kampala, Uganda; 7 Department of Global Health and Development, London School of Hygiene and Tropical Medicine, London, UK; 8 Department of Population Health, Wellcome Trust, London, UK

**Keywords:** Global mental health, Community mental health, Service user involvement, Peer support

## Abstract

**Background:**

Reducing readmissions among frequent users of psychiatric inpatient care could result in substantial cost savings to under-resourced mental health systems. Studies from high-income countries indicate that formal peer support can be an effective intervention for the reduction of readmissions among frequent users. Although in recent years formal peer support programmes have been established in mental health services in a few low- and middle-income countries (LMICs), they have not been rigorously evaluated.

**Methods:**

This protocol describes a quasi-experimental difference-in-differences study conducted as part of a broader evaluation of the Brain Gain II peer support programme based at Butabika National Referral Hospital in Kampala, Uganda. The primary objective is to investigate whether frequent users of psychiatric inpatient care who have access to a peer support worker (PSW+) experience a greater reduction in rehospitalisation rates and number of days spent in hospital compared to those who do not have access to a peer support worker (PSW-). Frequent users, defined as adults diagnosed with either a mental disorder or epilepsy who have had three or more inpatient stays at Butabika over the previous 24 months, are referred to Brain Gain II by hospital staff on five inpatient wards. Frequent users who normally reside in a district where peer support workers currently operate (Kampala, Jinja, Wakiso and Mukono) are eligible for formal peer support and enter the PSW+ group. Participants in the PSW+ group are expected to receive at least one inpatient visit by a trained peer support worker before hospital discharge and three to six additional visits after discharge. Frequent users from other districts enter the PSW- group and receive standard care. Participants’ admissions data are extracted from hospital records at point of referral and six months following referral.

**Discussion:**

To the best of our knowledge, this will be the first quasi-experimental study of formal peer support in a LMIC and the first to assess change in readmissions, an outcome of particular relevance to policy-makers seeking cost-effective alternatives to institutionalised mental health care.

## Background

Frequent users of psychiatric inpatient care, sometimes referred to as “revolving-door”, “high-frequency” or “heavy” users, consume a disproportionate amount of the limited resources available for mental health care [1, 2]. A systematic review of mostly high-income country (HIC) studies estimated 10–30% of users of psychiatric care consume 50–80% of service resources [3].

More recent studies from low- and middle-income countries (LMICs) observe high rates of readmission and large numbers of frequent users among psychiatric inpatient populations [1, 4–9]. In Nigeria, for example, 41.4% of psychiatric inpatients at a university teaching hospital were readmitted within five years. Among those readmitted, mean number of admissions was 2.9 [6]. Meanwhile, the average cost of a single admission to a Nigerian psychiatric hospital is $3675 USD, equivalent to the cost of 90 outpatient visits [10]. Reducing readmissions among frequent users could result in substantial cost savings to under-resourced mental health systems in LMICs.

While there is very little LMIC research investigating service user perspectives on readmission, it is generally acknowledged that readmission can be a profoundly disruptive and demoralising experience [11]. In overstretched psychiatric facilities, experiences of inpatient care may be particularly distressing. Human rights watchdogs have documented overcrowding, unsanitary conditions, abusive practices and other human rights violations at psychiatric inpatient facilities in a number of LMICs [12–16]. According to a survey of people with mental health conditions in LMICs, psychiatric facilities are the fourth worst setting in terms of human rights violations; prisons, by comparison, are sixth [17].

There is evidence from HICs that formal peer support can reduce readmissions [18–21]. Notably, a randomised controlled trial conducted in the United States showed that frequent users receiving formal peer support in addition to standard care had an average of 0.64 fewer readmissions and nine fewer days in hospital than those receiving standard care alone [22]. It is unknown whether similar outcomes can be expected in low-resource settings, as patterns in utilisation of inpatient care can differ substantially from those in high-income countries [23]. There have been no studies to-date on the effectiveness of formal peer support as an intervention to reduce readmissions in a LMIC setting.

Broadly defined, peer support is social emotional support that is mutually offered or provided by “peers”, people with lived experience of mental, neurological or substance use disorders [21, 24]. While peer support encompasses a wide range of different interventions, distinctions are made between formal peer support and informal peer support (or “naturally occurring” peer support, as described by Repper and Carter [2011, pp.393]) [24, 25]. Formal peer support is provided through peer-led programmes or by peers recruited to support roles in traditional mental health or social services [25]. Those offering formal peer support may refer to themselves as “peer support workers” (PSWs) [26], though peer support roles can vary greatly. (For example, the American trial described above employed “peer mentors” to deliver formal peer support [22].) Regardless of their role specifications, PSWs are generally considered to be further along on the road to recovery—able to manage their illness and pursue fulfilling lives—and thus able to leverage their personal experience of recovery to support others [18, 19, 24, 25, 27, 28].

The study described in this protocol is part of a broader evaluation of the Brain Gain II project in Uganda, one of the first LMICs to establish a formal peer support programme [29–31]. The aim of this study is to understand the impact of a formal peer support intervention delivered by trained PSWs on service users’ readmissions. The objective is to investigate whether frequent users of inpatient care who have access to peer support (PSW+) experience a greater reduction in rehospitalisation rates and number of days spent in hospital compared to those who do not have access to peer support (PSW-).

## Methods

As this is not a randomised trial, we first developed our protocol in accordance with STROBE (Strengthening The Reporting of Observational Studies in Epidemiology) guidelines [32], and then checked it against Reeves and Gaus’ (2004) adaptation of the CONSORT (Consolidated Standards of Reporting Trials) checklist for non-randomised studies [33]. Other components of the Theory of Change-driven evaluation have been protocolised and described elsewhere [34, 35]. These include: a cross-sectional survey of recovery-related knowledge, attitudes and practices among Butabika staff; a cost analysis to estimate money saved as a result of reduction in readmissions; a multi-method process evaluation; and additional qualitative methods comprising focus groups and interviews with study participants, PSWs, Butabika staff and other key stakeholders of the Brain Gain II project.

### Setting

Butabika National Referral Hospital (“Butabika”) is a tertiary psychiatric facility with approximately 430 staff and 550 beds [36], though the number of inpatients often exceeds 750 and can approach nearly 1000 [15]. Butabika is located in a largely suburban area of southeastern Kampala, but patients from across Uganda access its services. Standard adult care consists primarily of on-site psychiatric and psychological interventions, and in some cases vocational training, as prescribed by hospital staff. Users in extremely vulnerable situations may be referred to a social worker for additional assistance. After discharge, users typically return to Butabika for outpatient services, attend one of four monthly community outreach clinics (each located within a 20 km radius of the hospital), or access mental health services at district hospitals closer to their homes.

Brain Gain II is a project of the Butabika Link in Uganda—a partnership between Butabika and the East London National Health Service Foundation Trust (ELFT) in the United Kingdom [31]. Brain Gain II aims to reduce the burden on inpatient care at Butabika by promoting recovery among service users on the hospital wards and following discharge. The two main components of Brain Gain II include: (1) establishing an on-campus Recovery College at Butabika, where people with lived and/or professional experience of mental or neurological disorders co-design and co-deliver a recovery-oriented training curriculum; and (2) offering formal peer support by trained PSWs to frequent users of psychiatric inpatient care at Butabika, on five hospital wards and in local communities in four districts [37].

The Butabika Recovery College (BREC) is located in the Community Recovery Team building adjacent to the Forensic Ward. Recovery Colleges are educational (as opposed to clinical) spaces that operate similarly to unaccredited adult education colleges, though with a focus on meaningful involvement of people with lived experience in all aspects of their functioning [38]. At BREC, people with lived experience (mostly PSWs) and people with professional experience (Butabika staff) co-deliver regular teaching sessions on recovery-related topics. Most teaching sessions focus on “what helps” and “what hinders” recovery, identified through a series of Recovery Listening Events held in Uganda by the Sharing Stories Group at the start of Brain Gain II [39]. However, BREC also hosts yoga, bead-making and other skills-based teaching sessions. Most students are current inpatients, though BREC is also open to outpatients, family members and hospital staff.

The Peer Support Office sits within BREC, and acts as the coordinating centre for both Recovery College trainers and PSWs. PSWs operate on five hospital wards, including the forensic ward, acute admissions wards (male and female wards), and long-stay rehabilitation wards (male and female wards). The four districts where PSWs carry out community visits include Kampala, Jinja, Wakiso and Mukono. These are located in the Central and Eastern regions of Uganda and within approximately two hours’ drive of Butabika. Communities in these districts are typically urban or suburban, and English and Luganda are widely spoken.

### Study design

In keeping with Brain Gain II’s emphasis on co-production, it was agreed that both PSWs and staff should be involved in the design and conduct of the project’s evaluation. “A theory of how and why an initiative works” that can be empirically tested (Weiss 1995, p. 86 cited DeSilva et al. 2014, n.page), theory of change is increasingly recognised as a useful tool for involving diverse stakheholder groups in evaluation design [40–42]. Two days of Theory of Change workshops were carried out at Butabika, convening PSWs, Butabika staff and representatives of ELFT. Through guided discussions facilitated by the first author, short-, medium- and long-term outcomes were backward-mapped onto a “pathway of change”, which was further refined in consultation with the project leads at Butabika and ELFT [see Additional file 1]. Indicators were assigned to each outcome and methods proposed to measure each indicator, in order to build up the evaluation design. Methodological decisions were made with a focus on feasibility, understanding that PSWs and staff would be responsible for much of the data collection. As this was funded as a project evaluation, with limited budget for research, there was little scope for hiring external data collectors with the time and specialist expertise required to administer complex measurement tools.

For the evaluation of user-level outcomes of peer support, it was not considered appropriate by stakeholders to adopt an experimental design, in which frequent users in extremely vulnerable situations who could otherwise benefit from peer support in their local communities might not receive PSW visits. Hence, a quasi-experimental difference-in-differences (DID) study design was proposed in which the comparison group consists of those who are referred to the PSW programme but live outside of the four districts that comprise its current catchment area, and therefore do not have access to formal peer support. This design was modelled on a previous evaluation of community-based rehabilitation for people with severe mental disorders in India [43], and compares the change in number of rehospitalisations and hospital days among frequent users who have have access to a PSW (PSW+) to that of frequent users who do not have access to a PSW (PSW-).

### Participants

#### Eligibility criteria

**Fig. 1:**
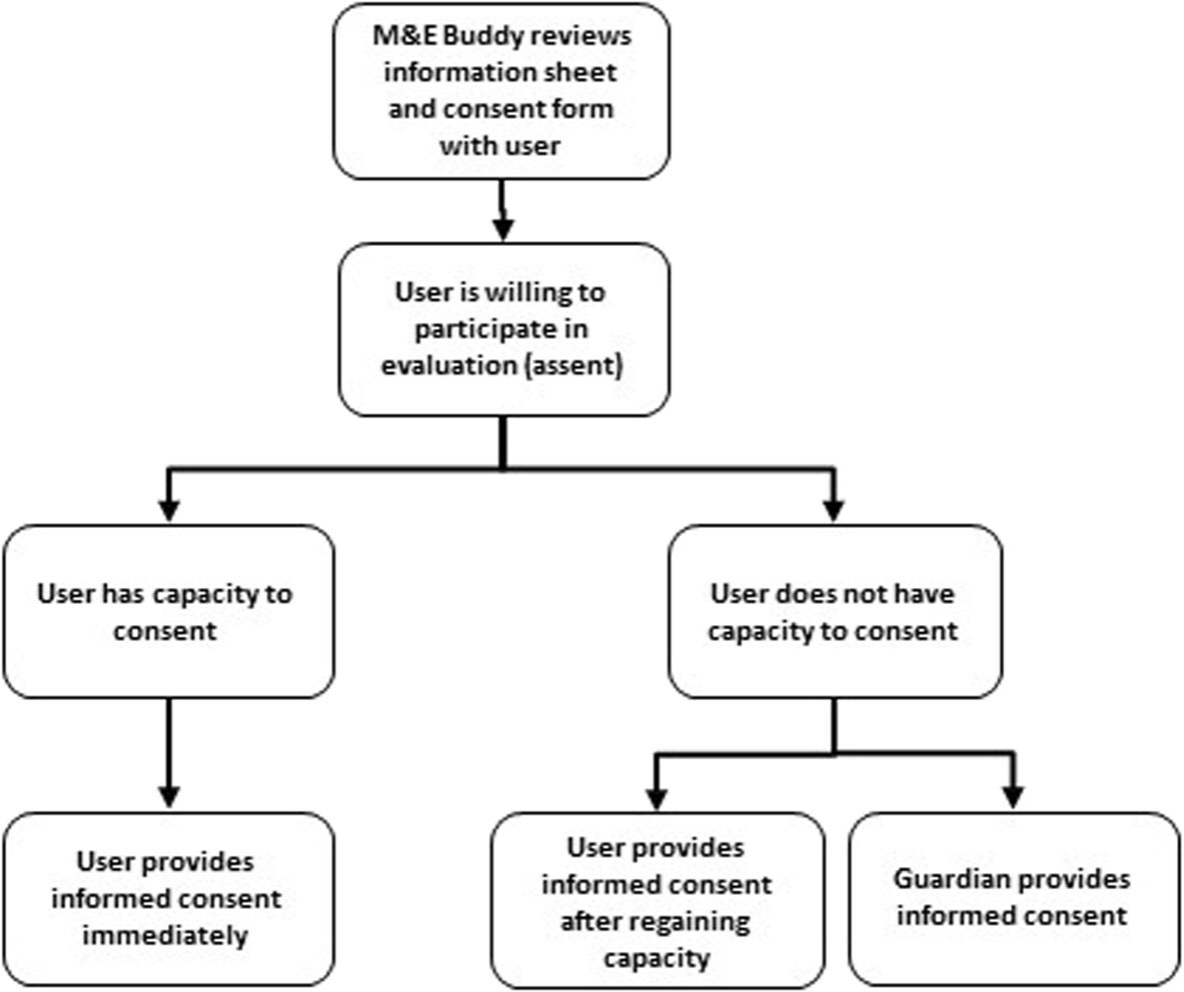
Flow chart for securing informed consent

In order to be eligible for inclusion in the study, a service user must: (1) be age 18 years or older; (2) be a Ugandan national currently residing in-country; (3) have been diagnosed with either a mental disorder or epilepsy; (4) have had three or more inpatient stays at Butabika in the previous 24 months; (5) be referred to the peer support programme from one of five participating hospital wards at Butabika; (6) agree to participate in the study (assent) at baseline; and (7) provide either informed consent or guardian consent within the six-month follow-up period (Fig. 1). Although Butabika’s Alcohol and Drug Unit is not one of the hospital wards participating in this study, service users with mental or neurological disorders who also have co-morbid substance use disorders will not be excluded.

#### Selection

Over a six-month recruitment period, staff from the five participating hospital wards will refer frequent users of inpatient care by completing referral forms with users’ demographic information, contact details, diagnoses, and admissions histories, extracted from patient records. As making referrals represents an additional unpaid administrative burden for already overstretched Butabika staff, Brain Gain II will offer a performance-based incentive to ward staff of 10,000 UGX (approximately $2.50 USD equivalent) for each referral form that is filled out completely and accurately. The Monitoring and Evaluation (M&E) Officer will review each form to confirm data quality before authorising a cash payment to be made directly to the staff member. Referral forms of sufficient quality will then be used to identify potential patients who meet the study criteria. Patients residing in Kampala, Jinja, Wakiso and Mukono will be eligible to receive the intervention, and patients residing elsewhere will form a comparison group.

#### Consent

The M&E Officer will assign specialised PSWs called “M&E Buddies” to visit potential participants on the hospital’s wards. M&E Buddies receive enhanced training in research procedures and ethics, including the use of the University of California, San Diego Brief Assessment of Capacity to Consent, a structured tool designed to assist research workers in assessing capacity to consent [44]. During the visit, a M&E Buddy will review the study information sheet and consent form with the participant in either English or Luganda and answer any questions.

All potential participants must assent before baseline data can be collected. Informed consent must also be secured in order for this data to be included in the evaluation. If the M&E Buddy suspects that a potential participant may not have capacity to consent, a guardian can consent as a substitute. Alternatively, a potential participant can provide informed consent at any point over a six-month follow-up period if he or she regains capacity. This provision is made to ensure that potential participants are empowered as much as possible to make their own decisions regarding participation. The process of securing informed consent is illustrated in Fig. 1.

### Peer support

All participants receive standard adult care and may have some contact with PSWs via the Recovery College. Additionally, participants in the PSW+ group receive face-to-face visits from a trained PSW. Each participant in the PSW+ group is assigned a PSW from a nearby community by an administrator in the Peer Support Office. Peer support visits are offered for up to six months after a PSW is assigned to a user. A recipient of peer support will be visited by a PSW at least once on the ward before discharge, and at least three times after discharge. For particularly vulnerable cases (i.e. three or more inpatient stays in the past 12 months), up to six visits can be made. The PSW visits occur per the recipient’s preference, either at home, at a meeting point in the community, or at the hospital when the recipient returns for outpatient visits. The carer may also be engaged in peer support visits, where possible.

The peer support visits delivered by PSWs are flexible and unstructured, and may consist of any or all of the following:

- **Befriending** (social contact, supportive listening and encouragement);
- **Role-modelling** (sharing personal experiences of illness and recovery);
- **Psychoeducation** (education on recovery principles);
- **Problem-solving** (discussing current challenges and possible solutions, liasing with providers to resolve issues with medical and social care as needed).

The qualifications, training, supervision and compensation of PSWs as well as quality assurance for peer support visits are detailed in Table 1.

**Table 1:** Brain Gain II Peer Support Workers

**Qualifications**	
PSWs must be adults (age 18+) with lived experience of mental or neurological disorders who are numerate, literate in at least one language and able to communicate in basic English. There is no minimum educational or professional qualification required to become a PSW.	
**Training**	
Thirty PSWs from communities in Kampala and nearby districts identified by the user-led organisation HeartSounds Uganda were trained in 2012, prior to the start of Brain Gain II. The five-day training was delivered in Kampala by three mental health professionals from the UK with experience managing peer support programmes. Training covered principles of peer support work, recovery and wellness, communications skills, techniques for managing aggression and using Tree of Life as a tool to positively reframe personal narratives of illness and recovery [31]. In March 2015 the trained PSWs participated in an additional Training of Trainers as part of Brain Gain II, to help build the capacity of new cohorts of PSWs [37]. The Training of Trainers has since been manualised and is available upon request.	
**Supervision**	
Group supervision is provided via Monthly Advisory Support Group meetings at Butabika. These meetings create opportunities for PSWs to discuss their work with one another and with Butabika staff, creating a forum for shared learning and problem-solving. If a particularly challenging medical or social issue is encountered, a PSW may request that a trusted staff member—usually a social worker or a nurse from Butabika’s Community Recovery Team—participate in the next visit. Monthly Advisory Support Group meetings are also opportunities to monitor the well-being of PSWs and provide additional support to those who are struggling. A PSW’s caseload may be redistributed to other PSWs from nearby communities, if necessary. A PSW recovering from a relapse is assessed by a psychiatrist at Butabika before resuming peer support visits.	
**Quality Assurance**	
At each visit, the PSW completes a structured follow-up form, which documents essential information such as the user’s up-to-date contact information and details about what took place. Forms are reviewed regularly by a M&E Officer to identify any inconsistencies which might suggest that a visit has not taken place, in which case an additional visit may be conducted by a Butabika staff member, to investigate.	
**Compensation**	
Although PSWs are not salaried hospital staff, they receive a lunch and travel stipend of 20,000 UGX (approximately $5 USD equivalent) for each day of activity.	

### Outcomes

For variables to be included in the primary analysis, Table 2 summarises the time-points for assessment, the form used and the data collector responsible.

**Table 2:** Assessment of Outcome Variables and Confounders for Primary Analysis

Variable type	Variable	Assessment	Time point	Data source	Method of Assessment
**Outcomes**	• Hospital days • Rehospitalisations	Baseline	Point of referral	Secondary data from paper-based records	Data extracted from patient file and entered into referral form by ward staff, then checked by M&E Officer
		Follow-up	Six months from referral	Secondary data from paper-based records	Data extracted from patient file and entered into six-month admissions form by M&E Officer
**Confounders**	• Disability • Family support	Baseline	Initial ward visit after referral	Primary data collected via questionnaire (based on WHODAS 2.0 and MIND ME)	Reported by user to M&E Buddy using baseline form

**Fig. 2:**
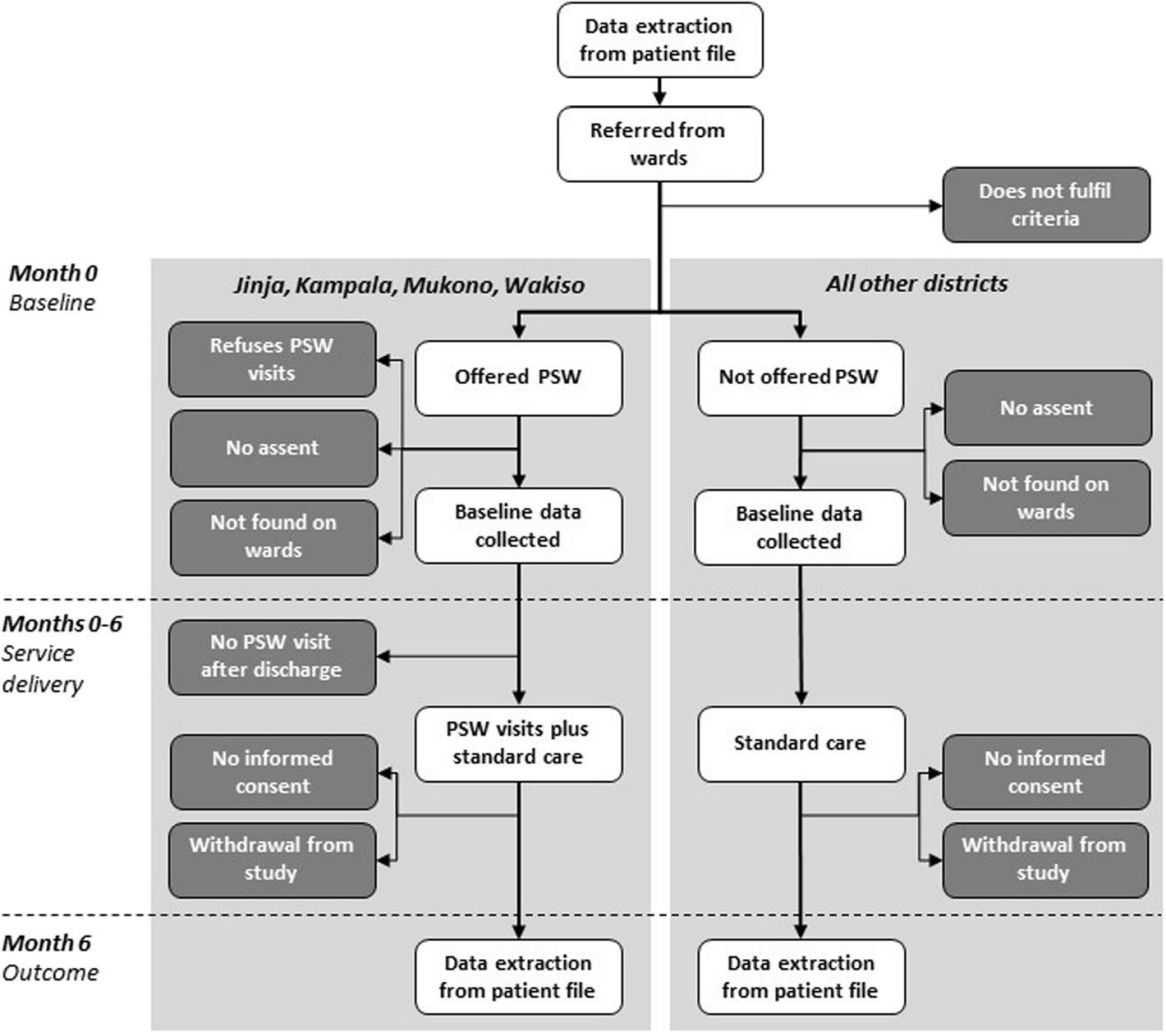
Flow chart for quasi-experimental study

There are two primary outcomes for this study, both related to change in frequent users’ utilisation of inpatient care at Butabika. The first is change in the number of hospital days over the previous six months. The second is change in the number of rehospitalisations over the previous six months. The index hospitalisation during which the participant was recruited into the study is excluded from both calculations. The study flow chart in Fig. 2 further illustrates the sequence of assessments in each group.

#### Hospital days

Hospital days are the number of days spent in psychiatric inpatient care at Butabika over the previous six months. In order to calculate the number of hospital days, dates of entry and departure are extracted from the patient file at the point of referral and six months from referral. An entry may be the result of formal admission or return to the hospital’s premises following an escape. Similarly, a departure may be the result of formal discharge or escape from the hospital’s premises.

#### Number of rehospitalisations

Number of rehospitalisations is the overall number of inpatient stays at Butabika over the previous six months. An inpatient stay is defined as a period of time spent in psychiatric inpatient care at Butabika and is also derived from the entry and departure data extracted from the patient file at the point of referral and six months from referral.

### Potential confounders

**Fig. 3:**
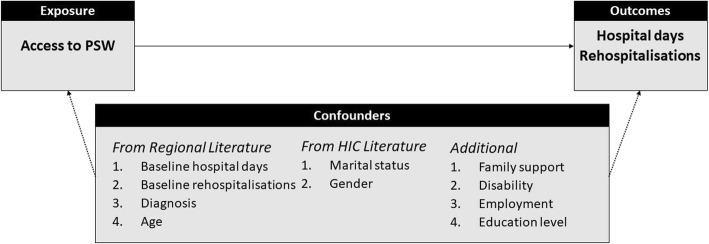
Initial working model

Our initial working model described in Fig. 3 suggests four confounders from previous research on risk of readmission in other sub-Saharan African countries [6, 45], two from HICs [46–50], and four proposed by the investigators: baseline values for the number of rehospitalisations and hospital days; baseline values for disability and family support; demographic factors, including gender, age, marital status, education level and employment; and diagnosis.

However, DID is designed to adjust for time-invariant and group-invariant confounders [51]. Age, gender, diagnosis, education level, employment and marital status are unlikely to change substantially between groups within the six-month follow-up period. We also observe cautions by Glymour et al. (2005) regarding adjustment for baseline measures of outcome variables (hospital days and rehospitalisations) [52]. Therefore, we plan to include only the remaining two proposed confounders (disability and family support) as pre-specified covariates in the adjusted analysis.

The M&E Buddy collects data on these two potential confounders by administering a baseline form to the study participant while he or she is still on the hospital wards [Table 2]. For those in PSW+ group, it is completed before any PSW visits take place.

#### Disability

Disability score is assessed using the 12-item World Health Organisation Disability Assessment Scale 2.0 (WHODAS 2.0) [53]. WHODAS originated as a tool for use in psychiatric inpatient settings. WHODAS 2.0 has been validated in diverse cultural contexts and settings, and is considered to be an acceptable cross-cultural measurement tool [53–55]. A Luganda version has been developed and used in primary care settings in Uganda by the PRIME research project [56]. We will use the unweighted “simple scoring” technique manualised by Ustun et al. (2010) to calculate disability scores from the WHODAS Likert scales.

#### Family support

Family support is measured using two separate three-item Likert scales: one for family’s attitude, and one for family’s willingness to help. Face validity of these scales was examined during the development of the Mental health Information aND Monitoring and Evaluation (MIND ME) Nigeria toolkit*,* and these measures have since been integrated into routine data collection for monitoring and evaluation of three Nigerian mental health programmes [57–59]. As with the “simple scoring” technique for WHODAS, we plan to sum these Likert scales in order to produce overall family support scores.

### Bias

Although this is not a blinded study, neither M&E Buddies, PSWs or the Peer Support Office Administrator responsible for assigning PSWs have access to participants’ admissions data. Admissions data are extracted from hospital records by ward staff at baseline and by a M&E Officer at follow-up [Table 2]. To reduce the risk of bias in the collection of additional data, a participant’s M&E Buddy will not also be assigned as his or her PSW.

### Study size

Brain Gain II aims to enrol 180 users into peer support over six months. Assuming at least a 15% refusal rate and 15% loss-to-follow-up [60], we expect a maximum of 126 participants to complete follow-up in the PSW+ group. With this estimate in mind, we conducted a sensitivity analysis exploring a variety of different scenarios relating to power, effect size and intraclass correlation, allowing for both balanced and unbalanced samples, at 0.05 alpha (see Additional file 2). Results suggest it is possible to detect a clinically meaningful effect (20% as per Cohen [1998]) with 80% power, or a larger effect (30%) with 90% power, if an additional 87 users are followed up in the PSW- group [61]. We therefore aim to recruit at least 129 users to the PSW+ and 101 to the PSW- group.

### Analysis

A detailed a priori statistical analysis plan has been drafted in consultation with a biostatistician and covers the baseline, primary and exploratory analyses described below. The plan will be finalised before any data are analysed and is available from the authors upon request. Any departures from the statistical analysis plan must be itemised and clearly justified in study reports.

We will use Stata/SE v15.1 for statistical analysis. All analyses will be performed at the level of the individual. Significance tests will be carried out with two-sided alpha of 0.05, and results will be reported with 95% confidence intervals.

#### Baseline

Descriptive statistics will be tabulated at baseline for both study groups (PSW+ and PSW-) [Table 3]. The mean, standard deviation and number of observations will be presented for all continuous variables. Numbers and percentages will be presented for categorical variables. Significance tests will not be performed to test for baseline differences between the study groups, as DID already presumes that study groups are unequivalent at baseline [51].

**Table 3:** Baseline characteristics for descriptive analysis

Variable type	Variable	Time point	Data source	Method of Assessment
**Demographic**	• Age • Gender • District of residence	Point of referral	Secondary data from paper-based records	Data extracted from patient file and entered into referral form by ward staff, then checked by M&E Officer
	• Education level • Occupational category	Initial ward visit after referral	Primary data collected via questionnaire (based on WHODAS 2.0)	Reported by user to M&E Buddy using baseline form
**Family**	• Family support • Marital status • Number of children	Initial ward visit after referral	Primary data collected via questionnaire (based on MIND ME and WHODAS 2.0)	Reported by user to M&E Buddy using baseline form
**Clinical**	• Diagnosis	Point of referral	Secondary data from paper-based records (based on MIND ME)	Data extracted from patient file and entered into referral form by ward staff, then checked by M&E Officer
	• Disability	Initial ward visit after referral	Primary data collected via WHODAS 2.0	Reported by user to M&E Buddy using baseline form
	• Years lived with mental health problem	Initial ward visit after referral	Primary data collected via questionnaire	Reported by user to M&E Buddy using baseline form
**Service use**	• Ward of referral • Hospital days • Rehospitalisations	Point of referral	Secondary data from paper-based records	Data extracted from patient file and entered into referral form by ward staff, then checked by M&E Officer
	• Previous Recovery College attendance • Satisfaction with hospital services	Initial ward visit after referral	Primary data collected via questionnaire	Reported by user to M&E Buddy using baseline form

#### Primary

The primary analyses will compare the six-month change in primary outcomes (hospital days and number of rehospitalisations) between the PSW+ and PSW- groups. We will carry out both intention-to-treat and per-protocol DID analyses for the two primary outcomes, presenting adjusted and unadjusted results [62]. To be included in the per-protocol analysis, participants in the PSW+ arm must receive at least one recorded ward visit and three recorded community visits during the six-month follow-up period [62]. As described above, disability and family support are potential confounders and will be included as covariates in the adjusted analysis.

We will use multivariable linear regression unless the distribution is skewed, in which case we may consider a Poisson or negative binomial regression, or another appropriate method. The impact of the peer support programme on hospital days and rehospitalisations will be estimated through mixed effects models with a random effect to account for correlations among users with the same PSW and fixed effects for access to peer support (PSW+ group versus PSW-group), time (baseline versus follow-up) and the interaction between access to peer support and time. The interaction estimating the change from baseline to follow-up in the PSW+ group relative to change in the PSW- group is the key effect of interest. We will tabulate the results at follow-up and differences from baseline by group with corresponding 95% confidence intervals.

#### Exploratory

Further exploratory analyses will be carried out to help contextualise the results of the main analyses and generate hypotheses for future testing. As primary data collection from the PSW- group is not possible at six-month follow-up, exploratory analyses are limited to the PSW+ group.

##### Disability, service satisfaction and family support outcomes

Likert scale data on disability, satisfaction with services and family support will be collected from the PSW+ group by M&E Buddies at both baseline and six-month follow-up. For both disability and family support, we will calculate summary scores at baseline and follow-up in the PSW+ group [53], then perform a one-sample paired t-test. If the data distribution is skewed, we will consider the Wilcoxon signed-rank test or another non-parametric alternative. For the five-item Likert scale question on service satisfaction, we will use chi-squared tests to test the significance of the difference between baseline and follow-up in the PSW+ group.

##### Other psychosocial outcomes

Retrospective data on a number of other psychosocial outcomes will be collected from the PSW+ group by M&E Buddies at six-month follow-up. These are categorical variables labelled “improved”, “no change” or “worsened”. We will present descriptive statistics showing proportion of participants in the PSW+ group who reported “improved” or “worsened” outcomes, for the following:

- Marriage or romantic relationship
- Parenthood
- Relationship with main caregiver
- Relationship with other family members
- Relationship with hospital staff (not PSWs)
- Social relationships (e.g. friends, neighbours)
- Work or income
- Education or training
- Housing
- Hobbies or recreation
- Physical health

#### Missing data

We expect attrition to be low, as the primary outcome data are derived from hospital records, meaning no follow-up contact with participants is required for the main analyses. The most likely reason for missing outcome data is loss of hospital records, which is not expected to affect the study groups differentially.

As per Schafer’s (1999) guidance, we will consider up to 5 % missing data to be inconsequential [63]. However, if more than 5 % of data is missing, then we will select appropriate principled missing data methods (e.g. multiple imputation), taking into consideration the data distribution and the mechanism, rate and pattern of missing data [64].

### Data quality

Although this study was not resourced to enable double data entry, we will undertake a number of other precautions to improve data quality. As described above, forms used for data collection are routinely checked for quality by the Brain Gain II M&E Officer, and performance-based incentives are also offered to staff for complete and accurate extraction of admissions data. Data validation rules are programmed into the spreadsheet used for data entry. We will carry out additional range and consistency checks during data cleaning.

At the conclusion of the six-month follow-up period, we will also perform a data quality audit. The primary investigator will sample every fourth participant file and check each paper form against a checklist for missing, illegible or illogical data. Data quality issues will be disaggregated by type and tabulated by data collector and question, to identify any common patterns. At the stage of data analysis, we will use either box-plots or z-scores to identify outliers. Where outliers are clearly the result of spurious data, corrections will be made if possible; otherwise, outliers resulting from spurious data may be treated as missing data.

## Ethics

This study was approved as part of a broader evaluation protocol for Brain Gain II. Institutional approval was received from the Research and Training Committee of the Butabika National Referral Hospital in Uganda. Ethics approval was secured from the London School of Hygiene and Tropical Medicine Research Ethics Committee in the United Kingdom (Ref 10,705) and Mengo Hospital Research and Ethics in Uganda (Ref 906/7). The evaluation protocol was also approved by the Uganda National Council of Science and Technology (Ref HS12ES). Additional details on the ethical considerations and procedures for this study are available from the study authors upon request.

## Discussion

This study will contribute to the evaluation of one of the first formal peer support programmes to be established in a LMIC. Given the high cost of inpatient care, the outcomes are particularly relevant to mental health policy in Uganda and other LMICs, where most government expenditure on mental health continues to be spent on psychiatric hospitals [23].

The use of a quasi-experimental study design is an improvement over previous evaluations of formal peer support in LMICs. Although formal peer support programmes have been established in statutory services in other LMICs such as China [65] and India [66], to the best of our knowledge, only one has published a quantitative evaluation of user-level outcomes. These outcomes were limited to change in mood and social communication skills, assessed retrospectively at a single time-point, with no comparison group [65, 67].

This is also one of remarkably few examples in which the manpower and unique expertise of people with lived experience of mental and neurological disorders is harnessed for the purposes of conducting evaluation research in a LMIC. A systematic review published in 2016 identified only one previous example; it came from Brazil, an upper-middle income country, and users were not involved until data had already been collected [68, 69]. A 2017 survey on psychosocial disabilities and barriers to participation in North India may have involved data collectors with psychosocial disabilities, although this is unclear from the study’s text [67, 70]. While engagement of M&E Buddies in data collection is desirable from an inclusion perspective, and may be more sustainable than relying on external evaluators, the use of M&E Buddies has not yet been tested in this context. Future publications will report not only on the outcomes of peer support in Uganda, but also on learning from the engagement of peers in the conduct of this study.

Unfortunately, a randomized control trial was not deemed acceptable for the purposes of this study. Indeed, researchers in HICs have pointed out that randomisation may be in opposition to the principles of self-determination embraced by peer support programmes [71], though some have carried out successful randomised-controlled trials nonetheless. DID is designed to control for time-invariant and group-invariant confounders, but does not entirely eliminate the possibility of confounding or other types of bias. As noted by Wing, Simon and Bello-Gomez (2018): “The DID design is not a perfect substitute for randomized experiments, but it often represents a feasible way to learn about causal relationships” (pp.453).

The study design outlined in this protocol introduces a risk of bias, as the two study groups differ by district of residence. There is a possibility that unmeasured time- and group-variant factors may differ between districts and confound the relationship between peer support and use of inpatient care. Peer support is only available in four districts of the relatively prosperous Central and Eastern regions near the country’s capital city, Kampala, where Butabika is located. Further, this is not a blinded study, though PSWs responsible for delivering the intervention do not collect study data from their assigned peers, and neither PSWs, M&E Buddies nor the Peer Support Office Administrator have access to admissions data.

In addition, the outcomes compared between groups are limited to those which can be assessed using the hospital’s routinely collected data. Loss-to-follow-up after discharge from inpatient care is a significant issue for Butabika, which PSWs help to remedy through regular visits to the community. The comparison group does not have contact with PSWs after discharge. Therefore, stakeholders deemed it unfeasible to collect primary data from a significant number of participants in the comparison group. The Indian evaluation upon which this study is based faced similar challenges; outcomes could not be assessed in about a third of all participants in the comparison group [43].

Finally, it is worth acknowledging that formal peer support can be challenging to implement [72]. For example, there is a risk that formalisation of peer support roles may actually reinforce hierarchical relationships in statutory services and ultimately undermine core peer support values [73]. At the same time, PSWs may be expected to serve as “carriers of a recovery culture into mental health systems” (Ibrahim 2019, n.page), resulting in conflict between PSWs and organisations resistant to change [72]. These are tricky relationships for PSWs to negotiate, even in relatively well-resourced settings. Meanwhile, resource limitations have been identified as major barriers to service user involvement in mental health systems strengthening in Uganda [74] and in LMICs more broadly [75]. While the Brain Gain I pilot demonstrated that it is feasible to deliver formal peer support in Uganda [31], there is always the possibility of “implementation failure” (Patton 2008, pp. 310) leading to null results [76]. In this case, our multi-method, Theory of Change-driven approach to the broader programme evaluation (described elsewhere [34, 35]) may help to identify “what went wrong” on the anticipated pathway of change and make targeted recommendations for future implementation.

In conclusion, this is a quasi-experimental DID study subject to a number of different constraints and potential biases, which should be followed up with more robust research, assessing more outcomes with locally validated measures, and ideally using a randomised design. Due caution will need to be taken in the interpretation of results. However, given the paucity of research currently available from LMICs, this study represents a crucial next step toward the development of a global evidence base for peer support.

## Supplementary information

**Additional file 1.** Brain Gain II Theory of Change map.

**Additional file 2.** Sensitivity Analysis for Primary Outcomes.

## Data Availability

Not applicable.

## Abbreviations

BELL: Butabika-East London National Health Service Foundation Trust Link
DID: Difference-in-differences
ELFT: East London National Health Service Foundation Trust
HICs: High-Income Countries
LMICs: Low- and Middle-Income Countries
M&E: Monitoring and Evaluation
MIND ME: Mental Health Information and Monitoring and Evaluation
PSW-: No Access to Peer Support
PSW: + Access to Peer Support
PSWs: Peer Support Workers
STROBE: Strengthening the Reporting of Observational Studies in Epidemiology
UGX: Uganda Shillings
USD: United States Dollars
WHO: World Health Organisation
WHODAS: World Health Organisation Disability Assessment Schedule
