# Supplementary material for: Peer support for frequent users of inpatient mental health care in Uganda: protocol of a quasi-experimental study
Source: BMC Psychiatry. 2019 Nov 29;19:374. doi: 10.1186/s12888-019-2360-8 (PMC6883561; doi:10.1186/s12888-019-2360-8)
Supplement: Supplementary file 1 — Additional file 1. Brain Gain II Theory of Change map. [file 12888_2019_2360_MOESM1_ESM.pdf]

Additional File 1: Brain Gain II Theory of Change Map

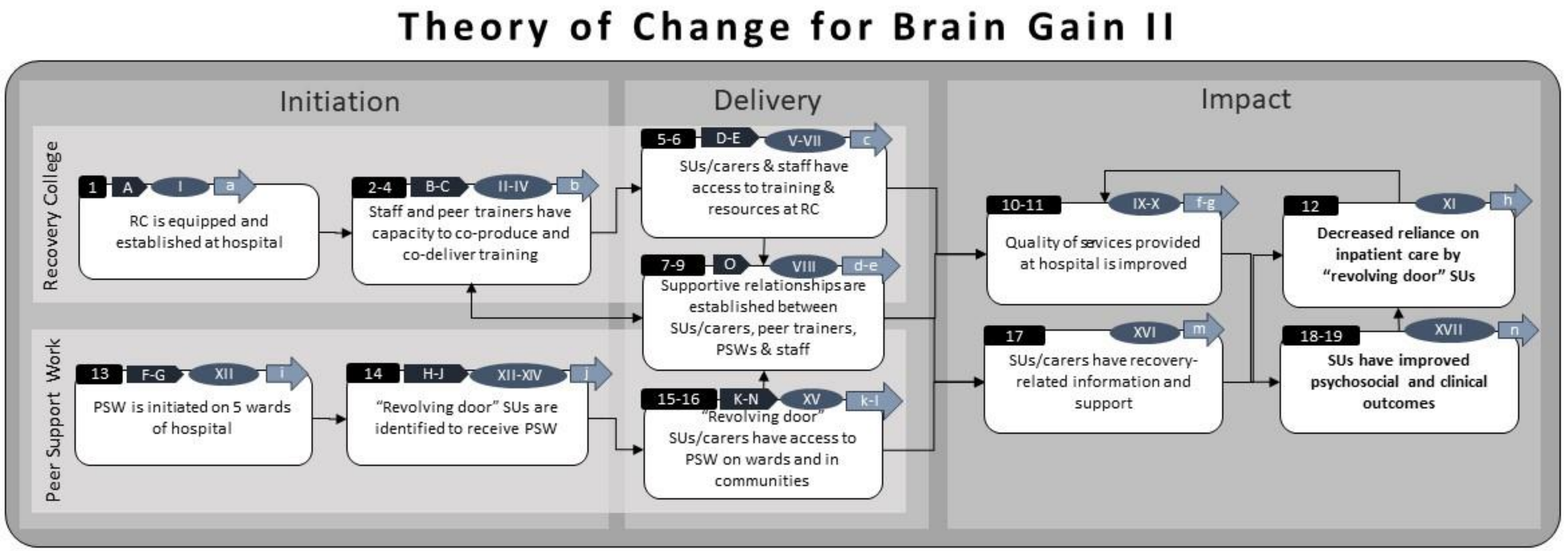

INDICATORS

1. Building refurbishment completed by target date
2. Number of attendees completing TOTs, disaggregated by attendee type
3. Proportion of attendees able to develop and execute a lesson plan during TOT demonstration
4. Mean improvement in peer assessment score between first and second TOT
5. Average number of RC trainings held per month after opening
6. Average RC training attendance, disaggregated by attendee type
7. Qualitative data collected from SUs, peer trainers and staff
8. Difference in family support scores at 6 month follow-up
9. Qualitative data collected from SUs, PSWs, staff and hospital administrators
10. Change in knowledge, attitudes and practices of hospital staff
11. Difference in patient satisfaction score
12. Change in number of inpatient days at 6 month follow-up
13. Proportion of identified wards accepting initial PSW visits
14. Average number of appropriate referrals to PSW per month, disaggregated by ward
15. Proportion of appropriate referrals receiving at least 1 ward and 3 community PSW visits
16. Average number of contacts with a PSW, disaggregated by contacts with SU alone, carer(s) alone, or SU and carers together
17. Perceptions of SUs and PSWs as discussed in focus groups
18. Difference in WHODAS 12-item disability score
19. Self-reported change in psychosocial circumstances, disaggregated by change in employment, education, relationships

RATIONALES

a. Location at hospital ensures accessibility and decreases stigmatization of Butabika
b. Co-production and co-delivery increase social contact between staff and peer trainers and role-model principles of equality to trainees, reducing stigma and building relationships
c. Creating a space for staff, SUs and carers to use increases social contact, reducing stigma and building relationships
d. Having relationships with SUs, peer trainers and PSWs encourages staff and carers to treat SUs as equals
e. PSWs add to SUs and carers social support network in the community
f. Quality services will better prepare SUs to manage in the community, decreasing likelihood of readmission
g. Quality services will improve outcomes of SUs leaving hospital
h. High patient load negatively impacts quality of care
i. Adult admissions wards are included except for drug and alcohol unit, as PSWs have not yet been trained to address addiction
j. "Revolving door" SUs are more likely to have issues managing their illness in the community, which PSWs aim to support
k. Having PSWs on wards increases social contact with staff, allows PSWs to help problem-solve when issues with inpatient care arise, and allows PSWs provide transitional support back to community at a point when SUs are particularly vulnerable
l. Having PSW in community helps to bolster social support network and problem-solve in the community, partially through interaction with carer
m. Adopting a recovery-oriented approach to care encourages illness management in the community and may improve outcomes
n. If outcomes are better, SUs will not require as much inpatient care

ASSUMPTIONS

I. Hospital staff and administration are willing and able to make a facility available for RC
II. Trainers are willing and able to collaborate
III. Trainers are willing and able to attend TOTs
IV. TOTs build sufficient capacity
V. Trainers are willing and able to produce and deliver regular trainings
VI. SUs, carers and staff are willing and able to attend RC without compensation
VII. Programme has sufficient human and material resources to keep RC open regularly for project duration
VIII. Interventions delivered through RC and PSW are sufficient to change relationships
IX. Interventions delivered and supportive relationships built through RC and PSW are capable of changing the quality of services
X. Reduction in client load from "revolving door" SUs is sufficient to enable hospital staff to take on quality improvement measures
XI. Better outcomes and access to quality services, information and support are sufficient to change illness management behavior
XII. PSWs from Brain Gain I are willing and able to deliver service on wards
XIII. Hospital staff and administration are willing to have PSWs on the wards
XIV. Ward staff are willing and able to identify and refer "revolving door" SUs
XV. PSWs, SUs and carers are willing and able to complete full course of visits
XVI. RC and PSW interventions are sufficient to equip SUs and carers
XVII. Information, support and quality services are sufficient to improve outcomes

INTERVENTIONS

A. Purchase equipment (furniture, computers, books, teaching supplies) and refurbish community building
B. Recruit staff from wards and peer trainers from existing PSWs and former peers from Brain Gain I
C. Conduct two TOTs for staff and peer trainers to attend collaboratively
D. Develop educational films on recovery and common questions about MNS disorders, as resources for RC
E. Conduct bi-monthly, co-produced and co-delivered trainings on recovery-related topics for a variety of attendee types
F. Recruit Brain Gain II PSWs from existing PSWs trained in Brain Gain I
G. Initiate mutual support groups for supervision
H. Conduct trainings of trainers for ward in-charges to learn to use referral forms
I. Ward in-charges train and supervise ward staff to make referrals
J. Monitoring and Evaluation Officer works with hospital staff to confirm "revolving door" status by examining patient records
K. PSWs are assigned correctly referred SUs in their respective catchment areas
L. PSWs conduct a minimum of 1 ward visit and 3 community visits to each assigned SU, interfacing with carers and ward staff as needed
M. During visits, PSWs role-model, educate on principles of recovery, aid in problem-solving, and/or offer encouragement, as needed
N. Ongoing monitoring and evaluation is carried out to confirm that SUs are receiving visits as intended
O. Staff accompany PSWs on visits for specialist assistance on an as-needed basis
