# Supplementary material for: Peer support for frequent users of inpatient mental health care in Uganda: protocol of a quasi-experimental study
Source: BMC Psychiatry. 2019 Nov 29;19:374. doi: 10.1186/s12888-019-2360-8 (PMC6883561; doi:10.1186/s12888-019-2360-8)
Supplement: Supplementary file 2 — Additional file 2. Sensitivity Analysis for Primary Outcomes. [file 12888_2019_2360_MOESM2_ESM.docx]

# Additional File 2: Sensitivity Analysis for Primary Outcomes

| Variable | Power | Alpha |  |  |  | **Receiving PSW Visits** | | | **Receiving Standard Care** | | | **Combined** |
| --- | --- | --- | --- | --- | --- | --- | --- | --- | --- | --- | --- | --- |
|  |  |  | ICC^[[1]](#footnote-1)^^[[2]](#footnote-2)^ | Ratio | Effect Size^[[3]](#footnote-3)^^[[4]](#footnote-4)^ | Mean^[[5]](#footnote-5)^ | SD | Sample | Mean | SD | Sample | Total Sample^[[6]](#footnote-6)^ |
| **Hospital Days** | 0.90 | 0.05 | 0.01 | 3:1 | 20% | 47.72 | 28.07 | 261 | 59.65 | 28.07 | 78 | 339 |
|  |  |  |  |  | 30% | 41.76 | 28.07 | **111** | 59.65 | 28.07 | **35** | **146*** |
|  |  |  |  | 2:1 | 20% | 47.72 | 28.07 | 192 | 59.65 | 28.07 | 88 | 280 |
|  |  |  |  |  | 30% | 41.76 | 28.07 | **82** | 59.65 | 28.07 | **39** | **121*** |
|  |  |  |  | 1:1 | 20% | 47.72 | 28.07 | 127 | 59.65 | 28.07 | 117 | 244 |
|  |  |  |  |  | 30% | 41.76 | 28.07 | **54** | 59.65 | 28.07 | **52** | **106*** |
|  |  |  | 0.04 | 3:1 | 20% | 47.72 | 28.07 | 396 | 59.65 | 28.07 | 78 | 474 |
|  |  |  |  |  | 30% | 41.76 | 28.07 | **126** | 59.65 | 28.07 | **35** | **161*** |
|  |  |  |  | 2:1 | 20% | 47.72 | 28.07 | 268 | 59.65 | 28.07 | 88 | 356 |
|  |  |  |  |  | 30% | 41.76 | 28.07 | **92** | 59.65 | 28.07 | **39** | **131*** |
|  |  |  |  | 1:1 | 20% | 47.72 | 28.07 | 164 | 59.65 | 28.07 | 117 | 281 |
|  |  |  |  |  | 30% | 41.76 | 28.07 | **59** | 59.65 | 28.07 | **52** | **111*** |
|  | 0.80 | 0.05 | 0.01 | 3:1 | 20% | 47.72 | 28.07 | 189 | 59.65 | 28.07 | 58 | 247 |
|  |  |  |  |  | 30% | 41.76 | 28.07 | **81** | 59.65 | 28.07 | **26** | **107*** |
|  |  |  |  | 2:1 | 20% | 47.72 | 28.07 | 140 | 59.65 | 28.07 | 66 | 206 |
|  |  |  |  |  | 30% | 41.76 | 28.07 | **60** | 59.65 | 28.07 | **29** | **89*** |
|  |  |  |  | 1:1 | 20% | 47.72 | 28.07 | **93** | 59.65 | 28.07 | **87** | **180*** |
|  |  |  |  |  | 30% | 41.76 | 28.07 | **40** | 59.65 | 28.07 | **39** | **79*** |
|  |  |  | 0.04 | 3:1 | 20% | 47.72 | 28.07 | 246 | 59.65 | 28.07 | 58 | 304 |
|  |  |  |  |  | 30% | 41.76 | 28.07 | **90** | 59.65 | 28.07 | **26** | **116*** |
|  |  |  |  | 2:1 | 20% | 47.72 | 28.07 | 176 | 59.65 | 28.07 | 66 | 242 |
|  |  |  |  |  | 30% | 41.76 | 28.07 | **66** | 59.65 | 28.07 | **29** | **95*** |
|  |  |  |  | 1:1 | 20% | 47.72 | 28.07 | **112** | 59.65 | 28.07 | **87** | **199*** |
|  |  |  |  |  | 30% | 41.76 | 28.07 | **43** | 59.65 | 28.07 | **39** | **82*** |
| **Rehospitalisations** | 0.90 | 0.05 | 0.04 | 3:1 | 20% | 1.32 | 0.24 | **27** | 1.65 | 0.24 | **8** | **35*** |
|  |  |  |  | 2:1 | 20% | 1.32 | 0.24 | **18** | 1.65 | 0.24 | **9** | **27*** |
|  |  |  |  | 1:1 | 20% | 1.32 | 0.24 | **12** | 1.65 | 0.24 | **12** | **24*** |

1. A protocol for the PREMIUM trial in India has used an ICC of 0.04 to estimate the therapist effect, but the authors note that this may be overly conservative, citing a HIC trial reporting within-therapist clustering <0.01. The sensitivity analysis floats ICCs of 0.01 and 0.04 for comparison, assuming 30 PSWs offer services. [↑](#footnote-ref-1)
2. Patel V, Weobong B, Nadkarni A, Weiss HA, Anand A, Naik S, Bhat B, Pereira J, Araya R, Dimidjian S *et al*: **The effectiveness and cost-effectiveness of lay counsellor-delivered psychological treatments for harmful and dependent drinking and moderate to severe depression in primary care in India: PREMIUM study protocol for randomized controlled trials**. *Trials* 2014, **15**:101. [↑](#footnote-ref-2)
3. A 20% mean difference is considered a small, clinically meaningful effect, and was included in the analysis for each of the two primary outcomes. For

   hospital days, a 30% difference was also considered for comparison. [↑](#footnote-ref-3)
4. Cohen J: **Statistical power analysis in the behavioral sciences**, 2 edn. Mahwah: Lawrence Erlbaum Associates; 1988. [↑](#footnote-ref-4)
5. Baseline values and standard deviations were estimated from Brain Gain II records of ten accurate referrals to peer support received in May 2015. A larger sample size could reduce the standard deviation, making this a conservative estimate. [↑](#footnote-ref-5)
6. Asterisk indicates a feasible sample size based on programme targets and assumptions. [↑](#footnote-ref-6)
